# dB/dt Evaluation in MRI Sites: Is ICNIRP Threshold Limit (for Workers) Exceeded?

**DOI:** 10.3390/ijerph15071298

**Published:** 2018-06-21

**Authors:** Giuseppe Acri, Patrizia Inferrera, Lucia Denaro, Carlo Sansotta, Elisa Ruello, Carmelo Anfuso, Francesca Maria Salmeri, Girolamo Garreffa, Giuseppe Vermiglio, Barbara Testagrossa

**Affiliations:** 1 Department of BIOMORF, University of Messina, Via Consolare Valeria, 98168 Messina, Italy; ldenaro@unime.it (L.D.); sansotta@unime.it (C.S.); eruello@unime.it (E.R.); fmsalmeri@unime.it (F.M.S.); btestagrossa@unime.it (B.T.); 2 School on Medical Physic, University of Messina, Via Consolare Valeria, 98168 Messina, Italy; patrizia.inferrera@gmail.com; 3 IRCCS Centro Neurolesi “Bonino-Pulejo”, Via Palermo, C.da Casazza, 98123 Messina, Italy; carmelo.anfuso@irccsme.it; 4 IEMEST (PA), Fondazione Potito (CB), FISMECO (RM), Via G. Donati, 00159 Roma, Italy; g.garreffa@gmail.com; 5 Department of P.A.S.S.I., University of Messina, Consolare Valeria, 98168 Messina, Italy; vermigli@unime.it

**Keywords:** static magnetic field, time-varying magnetic field, MRI safety, MRI workers exposure

## Abstract

The Directive 2013/35/EU establishes standards for workers exposed to static and time varying magnetic fields. These limits are based on ICNIRP guidelines expressed in terms of the electric field induced in the body. The complexity of this measurement led to theoretical models being developed. In this study, the experimental evaluation included varying magnetic field exposures for two classes of MRI workers. The measurements are conducted on four different MRI Systems including one 0.35 T, two 1.5 T, and one 3.0 T. Pocket magnetic dosimeters were used and it was carried out during routine conditions, emergency conditions, and cold-head maintenance/substitution. The acquired data has been processed and the corresponding dB/dt curves have been computed as the first time derivative of the dataset. The weighted peak approach was also implemented for the compliance assessment with regulatory limits. The dB/dt peak values have been compared with the reference level (RL) proposed by ICNIRP. The results show that the RL always exceeds during measurements on the 3.0 T scanner and sometimes on 1.5 T. In light of the foregoing, the diffusion of ultra-high field MRI scanners involves the introduction of behavioral rules that could be more useful than a numerical action level.

## 1. Introduction

Magnetic Resonance Imaging (MRI) is an excellent diagnostic non-invasive technique that provides images of internal tissues without applying ionizing radiation [1,2,3].

MRI scanners combine three different magnetic fields including the static magnetic field (typically symbolized by B_0_), the radiofrequency (RF) field (B_1_), and the magnetic field gradients in the three spatial directions (dB/dx, dB/dy and dB/dz). The magnetic field gradients are switched on and off to select the region of interest for spatial encoding of the image.

The possibility that some hazards may be associated with performing MR diagnostic images has been of concern ever since the introduction of this technique in the late 1970s [4].

The static magnetic field is used to align the nuclei in patients’ body. This powerful static magnetic field is always present even when the MRI scanner is not imaging [5]. There are two major safety issues regarding the static magnetic fields used in MRI: (a) translational force acting on ferromagnetic objects and a significant torque applied to objects with highly asymmetric shapes [6] and (b) direct interaction between the static magnetic field and living tissue [6]. The static magnetic field interacts with molecules and causes diamagnetic susceptibility anisotropy (DSA) since it brings elements to the magnetization of human cells that are subjected to a rotational torque [7] and magnetohydrodynamics, which is a macroscopic effect that involves moving charged objects in large organized structures such as DNA and proteins [7]. In addition, electrodynamic interactions with ionic conduction currents and effects on electronic spin states of the reaction intermediates in charge transfer processes are well established [8].

It is well known that movement in a magnetic field can induce a voltage in electrically conductive materials including biological tissues [6]. This can cause both sensory effects such as nausea, vertigo, etc. and stimulation of peripheral nerve due to the presence of eddy currents, which are related to a low electric field generated by motion.

RF pulses are used in MRI for nuclei excitation and interact with patient tissues causing heat. The rate of RF deposition is well determined in terms of the Specific Absorption Ratio (SAR). The effects caused by this kind of field decrease when moving away from the transmit coil [1]. In general, occupational exposure to the RF field is low since the field falls off rapidly outside the transmit coil. However, the healthcare staff could be exposed to the RF field during interventional procedures to levels similar to those experienced by the patient or volunteer undergoing the procedure [1].

When magnetic fields change with time in strength or direction, they can induce electric currents. The gradient coils in MRI systems during the switching mode can generate electrical potentials on a non-moving subject. Therefore, time-varying gradient fields can induce electric fields that create eddy currents in biological systems [7].

When the currents are strong enough, they can cause effects on the Central Nervous System (CNS) and also stimulate peripheral nerves. These effects range from almost imperceptible up to annoyance and pain in the case of peripheral nerve stimulation and cardiac fibrillation [9].

The combination of these fields, acting directly on patients, can produce several but not serious physiological diseases [7,8,10,11]. Fortunately, these side effects do not occur every time and for all patients [9,12].

MRI staff is exposed to static magnetic field and, occasionally, to radiofrequency field and time varying gradient fields. In this context, occupational staff members stay in areas where the magnetic field changes steeply. For this reason, it is necessary to guarantee the safety of all the involved subjects that have to work within the MRI site including engineers for maintenance activities, nurses who attend patients with special needs while the examination is going on, cleaners, and radiographers.

In this regard, the effect of magnetic fields on the health care staff is still being studied because of the prolonged periods of time they spend near the MRI system [13,14]. Data suggest that acute effects suffered by the staff are associated with the combination of time varying magnetic field (dB/dt) and the strength of the static magnetic field B_0_ [15] particularly during patient positioning and removal [16]. In this context, it is also important to assess the MRI staff exposure to time varying magnetic field. In fact, workers moving through the magnetic field will experience time-varying current flow in the body as a result of their movement.

Due to this concern, the EU and ICNIRP have defined workers’ exposure limits to the risks arising from physical agents (electromagnetic fields) [17,18,19].

The objective of ICNIRP guidelines [19] is to prevent peripheral nerve stimulation and to minimize the possibility of transient sensory effects as a consequence of electric field induced in the human body by movements in static magnetic fields within occupational setting.

The ICNIRP guidelines specify the differences between “basic restrictions” and “reference levels.” “Basic restrictions” on exposure to magnetic fields are based on established adverse health effects while “Reference Levels” (RL) are values provided for practical exposure assessment purposes to determine whether the basic restrictions are likely to exceed. Compliance with the RL is designed to ensure compliance with Basic Restrictions.

In order to prevent sensory effects such as vertigo and nausea arising from motions in a static magnetic field, the ICNIRP guidelines [19] and the ICNIRP Fact Sheet [20] establish basic restrictions. They also state that the change of magnetic flux density ΔB should not exceed 2 T during any 3 s period for relatively slow motions in a static magnetic field while, to prevent peripheral nerve stimulation arising from fast transient motions, the peak internal electric field should not exceed 1.1 V/m. For this reason the probability of vertigo and nausea should be low if it is possible to move so slowly that the maximum ΔB does not exceed 2 T during any 3 s period [19].

A practical way for determining if compliance with the basic restrictions for fast transient motions exists is to ensure that the magnetic flux density does not exceed the RL derived conservatively from the basic restrictions. In order to avoid electrical stimulation of peripheral nerves, the RL for peak dB/dt has been set to 2.7 T/s while, to prevent magnetophosphenes, it is recommended to limit the internal electric field below 0.7/*f* and peak dB/dt below 1.8/*f* (*f* is the frequency in Hz) [20]. Since the limits for the magnetophosphenes change as a function of frequency, it is necessary to apply weighted peak procedures explained in the ICNIRP 2010 guidelines [18].

The European Parliament in its Directive 2013/35/EU establishes a delegated act in order to insert the ICNIRP Guidelines for limiting exposure to electric fields induced by movement of the human body in a static magnetic field and by time-varying magnetic fields below 1 Hz into Annex II [17]. In order to facilitate the implementation of this Directive, non-binding practical guides are available. In Volume 1, following the art. 10 of the EU Directive, a conditional derogation from the requirement to comply with the exposure limit values is granted. Moreover, no limit value referring to occupational movement inside the scanner room is tabulated [21]. The Volume 2 of the non-binding guides good practice for implementing the EMF Directive (2013/35/EU), which deals with case studies for a range of occupational sectors, but it does not take into account the MRI healthcare staff and the spatial changing of the magnetic field during the operators’ motion [22].

A number of studies assessing the health risk for MRI workers has been published, which highlights the occurrence of transient symptoms induced by worker movements in the fringe field such as dizziness, vertigo, nausea, tinnitus, and concentration problems [12,23].

The direct measurement of the electric field and currents induced in the body is not a trivial task. For this reason, theoretical models for the evaluation of electric fields and current densities induced in the whole body in male and female workers during motion around the magnet have been developed and presented in References [16,24,25,26]. In particular, Sannino et al. developed [26] a numerical tool in order to evaluate the exposure to time-varying electric fields induced by motion. The time-varying electric fields were evaluated from a Matlab script, which starts from measurements conducted using a gaussmeter and observing workers’ motion inside the magnet room.

At the present time, in order to perform a time varying magnetic field due to body motion inside the magnet room, no standardized assessment procedures and methodologies are available in the literature.

This issue has been discussed elsewhere. In Reference [27], the exposure to static magnetic fields has been evaluated in sites that used MRI scanners ranging from 0.25 T up to 3.0 T. Later, in Reference [28], the computation of the dB/dt value exclusively on a 3.0 T MRI system has been developed.

In recent studies about the occupational exposure to static magnetic fields [29,30,31], the reported results were in accordance with those obtained in Reference [27].

The aim of this study is the implementation of a new methodology in order to compute the time varying magnetic field exposure, which starts from the magnetic flux densities measured by pocket personal magnetic dosimeters. The use of these dosimeters doesn’t impair working ability so that they can correctly reproduce the movement of the healthcare staff during their daily activities of positioning and/or removing patients.

The proposed experimental methodology is based on computation of the first time derivative of the magnetic flux densities data (dB/dt). The corresponding dB/dt peak values have been compared with the ICNIRP Guidelines RL [19]. At the same time, the weighted peak indexes for sensory (WP_L_) and health (WP_H_) effects were evaluated in order to assess if compliance with ICNIRP 2014 and EU Directive existed. This methodology can be useful for evaluating workers’ exposure referring to long-term neurobehavioral effects, which are yet unknown.

The measurements were conducted on different MRI Systems ranging from 0.35 T to 3.0 T and performed using personal dosimeters worn by the healthcare staff during patient positioning/removing and during emergency simulations. One of these dosimeters was also worn by a manufacturer technician during the maintenance of the cold head of a 3.0 T MRI scanner.

## 2. Materials and Methods 

Personal time varying magnetic exposure measurements were computed in three different contexts: (a) routinely health care staff working; (b) simulating an emergency condition; and (c) cold head maintenance/substitution performed by a manufacturer technician on a 3.0 T scanner.

To conduct measurements of a time-varying magnetic field, two Talete System Magnetic Dosimeters (TSMD—Tecnorad^®^ Verona, Italy) named TSMD_1 and TSMD_2, respectively, were used. Both the dosimeters consist of a Personal Monitoring Unit (PMU) coupled with a dedicated software and work in the 0 < B < 3 T range with a DC measurement sensitivity of 1 mT and an accuracy of ±0.05 mT. These PMUs are pocket devices that can be worn by MRI operators and are able to measure and store magnetic flux density values.

Both the TSMDs were calibrated directly by a manufacturer. In order to ascertain if differences in the results occur, we set two different sampling rates. A sampling rate of 0.2 s represents the standard set value, which has been set for TSDM_1 while, following the authors indications, a sampling rate of 0.056 s has been set for the TSDM_2. The 0.056 s sampling rate is the smallest value that can be set by the manufacturer.

The TSMD_1 and TSMD_2 were simultaneously worn by the MRI operators. The first time, both the dosimeters were placed on the operators’ torso and, on the second one, they were positioned on their head. In fact, the head represents the most likely target organ for reported neurobehavioral effects [32] and its swaying is greater than the torso one.

The measurements related to the contexts (a) and (b) were performed on four different MRI systems, which is one permanent magnet (Siemens 0.35 T) and three superconductor scanners (Siemens 1.5 T, Philips 1.5 T, and 3.0 T) during patient positioning operations and simulation of an emergency, which includes the rapid movement of the healthcare staff inside the magnetic room to assess the dB/dt values. These measurements were repeated three times and the dosimeters were worn by twelve workers who were different in age, size, and behavior inside the MRI room. All the involved subjects were MRI technicians.

Regarding the measurement carried out in the context (c), in order to perturb the work activities as little as possible, the technician wore only the TSMD_1 on his torso during the whole cold-head maintenance period. The cold-head is an important and critical mechanical component of the superconductor MRI scanner. Actually, the MRI superconductor systems require a cooling close to the absolute zero point in order to avoid thermal losses due to the extremely high currents in the coils of the electromagnet used to generate the static magnetic field [33]. This kind of measurement was conducted only once because the cold head maintenance represents an out of ordinary technical action.

The data were acquired by the TSMDs in terms of the function B = B(t) related to the movement of operators inside the MRI room. The stored data were then transferred on a PC and processed using Origin Data Analysis Software (Microcal Software INC., Northampton, MA, USA) to compute the corresponding dB/dt curves that were calculated by taking the time derivative of the experimental data. In practice, Origin treated discrete data by transforming the centered difference formula and calculated the derivative at each point of the B = B(t) function by taking the average of the slopes between the point and its two closest neighbors. This approach represents the best linear approximation of the function near the input value and it sounds as the “instantaneous rate of change.”

In order to compare the peak dB/dt with the ICNIRP RL and EU Directive action levels, we derived dB/dt action levels using the following equation, which was proposed by Mc Robbie [34].

(1) $$\;{\left(\frac{\mathrm{dB}}{\mathrm{dt}}\right)}_{peak}=\sqrt{2}\times 2\pi f\times B_{L}$$

As proposed in the EU Directive, the root mean square (RMS) high action level computed as 3 × 10^5^/*f* µT in the frequencies of 1 Hz–3 kHz corresponds in the dB/dt peak level value of 2.66 T/s. In the above frequencies, the range of the dB/dt peak value doesn’t depend on frequency.

For this reason, in order to avoid electrical stimulation of peripheral nerves, the ICNIRP proposed as RL for peak dB/dt has a value of 2.7 T/s. According to the ICNIRP guidelines and the EU Directive, we also compute the weighted peak indexes, which represents the observed exposure divided by the limit value. For practical purposes, the flux densities data B = B(t) are post processed using Origin Data Analysis Software in the frequency domain. In order to compute the WP_s_, a discrete Fourier Transform is performed on the stored data by applying the proper weighted factor and phase shift to each spectral component [21]. The exposure indexes represent The WPL and WPH, which are compliant when their values are less than one.

## 3. Results

**Figure 1:**
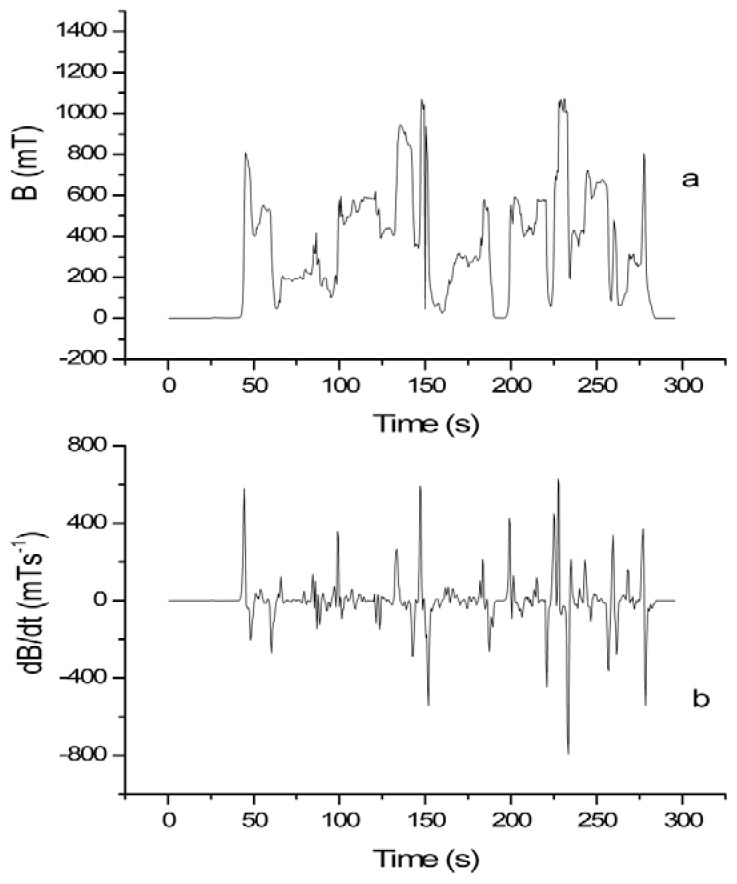
The measurement related to the TSMD_2 dosimeter, used on a 1.5 T site, and placed on the MRI technician’s torso. The data were recorded during patient positioning. (**a**) the exposure is shown; (**b**) the corresponding determined dB/dt curve is represented.

In Figure 1, the results of the TSDM_2 measurements, which are conducted on a 1.5 T MRI scanner, are presented. In this case the dosimeter was placed on the MRI technician’s torso and the data were recorded during patient positioning. Figure 1a shows the exposure as a function of time while Figure 1b reports the dB/dt curve obtained as the first time derivative of the experimental acquired data of Figure 1a.

**Figure 2:**
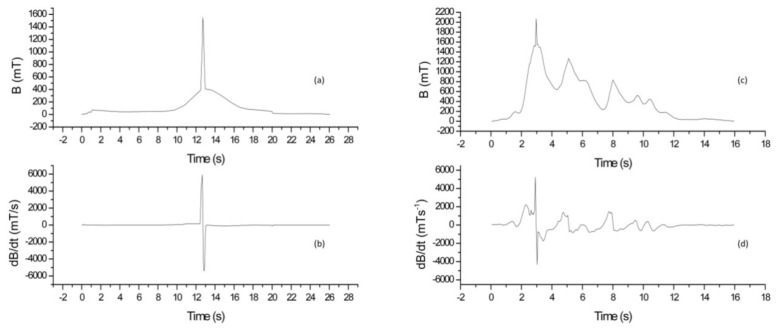
Measurement related to the TSMD_2 dosimeter, used on a 3.0 T site, placed on the head of two MRI technicians. The data were recorded during a simulation of an emergency. (**a**,**c**) the exposure is shown and (**b**,**d**) the corresponding computed dB/dt curve is represented.

In Figure 2, the results of the TSDM_2 measurements, which were conducted on the 3.0 T MRI scanner during an emergency simulation, are reported. In Figure 2, two MRI technicians who have different ages, sexes, sizes, and levels of experience are considered. In both cases, the dosimeter is positioned on the operators’ head and the dB/dt values are evaluated during patient removing procedures. In particular, Figure 2a,b refer to a female (worker 1) while, in Figure 2c,d, the MRI technician is a male (worker 2). The comparison between Figure 2a,c and Figure 2b,d highlighted the differences in the behavior of the MRI technicians. First of all, the time required for patient removing procedures was different, i.e., 26 s and 16 s for worker 1 and worker 2, respectively. In addition, worker 1 experienced a higher dB/dt peak value than worker 2 (5926.98 mT/s versus 5340.42 mT/s). In all cases, the experimental data confirm that workers with the same job experienced different exposure levels to the time varying magnetic field.

All the results of the measurements performed using the TSMDs on different MRI scanners during patient positioning and in emergency conditions are reported in Table 1 and Table 2. The absolute values of the dB/dt peaks are calculated when the mean value of the maximum dB/dt peak values are obtained for each operator during routine or emergency procedures.

The mean values of the absolute dB/dt peaks are reported with uncertainty. For the computation of the uncertainties, the maximum absolute error has been considered while the “duration” has been reported without uncertainty. Only the minimum and maximum time durations of patient positioning/removing activities in normal and emergency conditions have been indicated. We point out that the durations of routine and emergency procedures were very different because of the different behaviors of the MRI technicians. In Table 1 and Table 2, the TSMDs positions are indicated.

**Figure 3:**
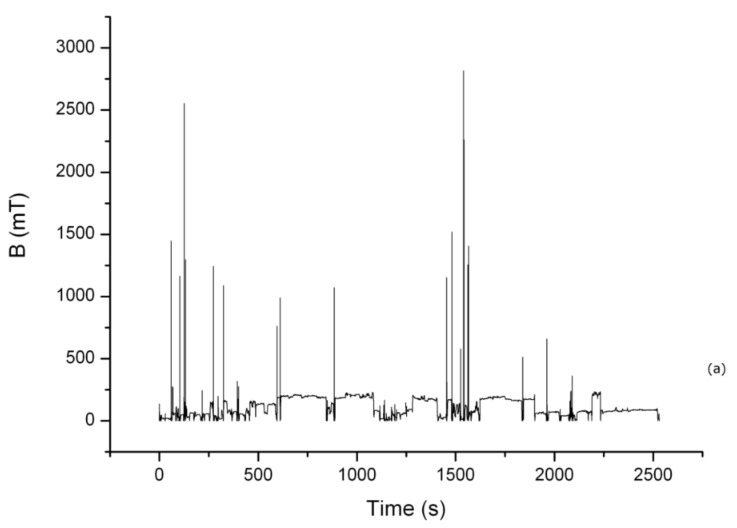

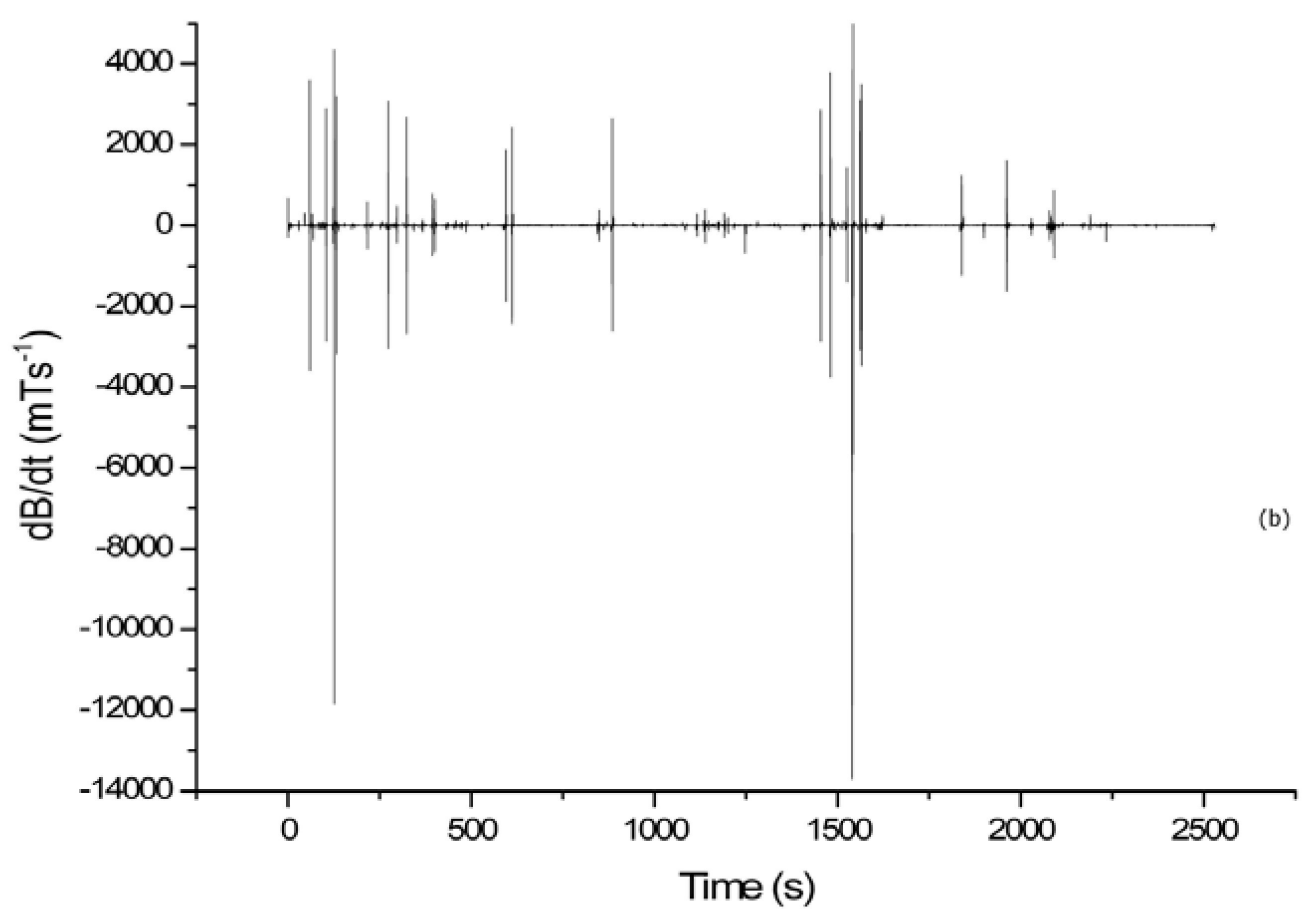
Measurement conducted in a 3.0 T site during cold head maintenance. (**a**) The worker exposure is reported and (**b**) the corresponding time-varying exposure is shown.

In Figure 3a, the measurement related to the TSDM_1, which is worn by the manufacturer technician during the cold head maintenance (about 43 min), is reported. In Figure 3b, the corresponding time varying magnetic exposure, dB/dt, is shown. In this case, only the TSMD_1 was used because the TSMD_2 sampling rate was four time shorter than the TSMD_1 one, but both the dosimeters had an equal mass storage. Therefore, the use of TSMD_2 was not allowed to conduct the measurement for the duration required for the complete maintenance operation.

In Table 3, we report the maximum peak value of the dB/dt obtained during the maintenance activity.

In Table 4 and Table 5, the computed dB/dt peak values and the WP_L_ and WP_H_ indexes are reported. In particular, the WP_L_ and WP_H_ indexes referred to the routinely procedures are indicated in Table 4 while the WP_L_ and WP_H_ indexes referred to the emergency simulation are shown in Table 5. In both tables, the minimum and maximum values of WPs obtained from the computation of repeated measurements conducted on each MRI scanner are reported. Bold values indicate noncompliance with the corresponding regulatory limits.

In Table 6, the computed dB/dt peak value and the WP_L_ and WP_H_ indexes, which are related to measurements conducted during cold head maintenance, are reported.

## 4. Discussion

In the current study, the time varying magnetic field exposure was evaluated using a different approach based on experimental evidence. Two classes of MRI operators were involved including the MRI technicians and the service technicians. The measurements were conducted on different MRI systems ranging from 0.35 T to 3.0 T. The dosimeters were worn by the MRI technicians on the torso and the head to assess the dB/dt peak value. All the obtained results were compared with the RL proposed by the ICNIRP Guidelines [19,20] and with the Action Levels stated in the EU Directive. The study also used the weighted peak indexes.

The analysis involving the healthcare staff was conducted by simulating normal and emergency conditions. It was demonstrated that the dB/dt peak values strictly depend on human motion through strong static magnetic fields. In this context, it is important to take into account that different clinical workers experience different exposures with exposure patterns also depending on patients (pediatric, in vegetative state, etc.). The measurements referred to the 0.35 T scanner are far from the RL both in normal and in emergency simulation.

The scenario changed when the measurements were conducted on the 1.5 T MRI devices. In these cases, when the dosimeters were worn on the operators torso, the dB/dt mean peak values during patient positioning were below the RL proposed by the ICNIRP guidelines while the dB/dt mean peak values obtained from emergency simulations were very close or slightly higher than the RL. In these cases, the Action Levels always surpassed. When the dosimeters were placed on the operators’ head, the results obtained from the dosimeter with the faster sampling rate (TSMD_2) during patient positioning were very close to the 2.7 T/s RL while, during the emergency simulation, this level was always higher.

The results of the measurements conducted on the 3.0 T MRI system highlighted that the RL of 2.7 T/s increased both in normal and emergency conditions when the dosimeters were placed on the operators’ head. However, when the dosimeters were worn on the MRI technicians’ torso, the obtained dB/dt mean peak value were very close to the proposed level.

The results referring to the healthcare staff are not in accordance with those reported by Zilberti et al. in Reference [16], which was obtained by using a theoretical model that simulates healthcare staff trajectories around the magnet. However, they are in accordance with Fatahi et al. [31], which were obtained with different strategies and instrumentation.

The results carried out from the measurements conducted during the cold head maintenance (context (c)) have highlighted a dB/dt peak value of 13,900 mT/s. In this case, the data were continuously collected for about 43 min. The time varying magnetic field peak value was extremely higher than the RL proposed by the ICNIRP.

At the end of the cold head maintenance, the technician reported vertigo and headache and he confirmed that these sensory effects manifested whenever he performed this kind of maintenance.

The results shown in Table 4 highlight compliance with the EU Directive Action Levels when the dosimeters were worn by operators on their torso while, when the dosimeters were positioned on the head of the MRI operators, the Low Action Levels are higher during measurements conducted on the 3.0 T. The results obtained during emergency simulation have shown that the WP_L_ indexes are greater than 1 in all the measurements conducted on the 3.0 T scanner especially when the TSMD_2 was placed on the operators head (the WP_H_ was greater than 1 in this case). Greater attention needs to be paid to the results reported in Table 6 and related to the cold head maintenance made by the manufacturer technician. In this case, both WP_L_ and WP_H_ are greater than 1. 

The data analysis and the comparison between the dB/dt peak values of different MRI technicians demonstrated that these peak values are strictly dependent on human motion through static magnetic fields. This motion dependence of the dB/dt was extremely evident when the TSMD_2 has been placed on the operators’ head. In fact, in this circumstance, the recommended RL proposed by the ICNIRP was always higher when the measurements were conducted on the 3.0 T MRI system while the results obtained from measurements conducted on the 1.5 T devices are very close to the RL. Comparable results are obtained using the weighted peak index method, which was suggested by the ICNIRP and EU Directive.

The developed methodology allowed us to estimate, in a quantitative manner, the dB/dt workers’ exposure at different MRI sites.

## 5. Conclusions

In conclusion, the MRI is considered by the research and medical community to be safe when compared to other more invasive neuroimaging modalities [24]. However, the MRI is not without risk. There are risks not only for patients but also for the healthcare staff when considering the imminent diffusion of extremely high field MRI scanners. In this context, the aim of this study was to conduct a risk assessment for MRI workers by introducing an alternative methodology based on experimental measurements in order to evaluate the dB/dt peak values that were obtained as the first time derivative of the experimental data. The availability and applicability of the proposed methodology has been tested on different MRI devices. The obtained results confirmed the flexibility of the method, its simplicity, and its reproducibility as an efficient tool to evaluate the dB/dt peak values. The measurements were conducted using two dosimeters with different sampling rates. Both the dosimeters were pocket size and wireless, which means they don’t hinder the operators’ movements. 

The obtained results were then compared with the ICNIRP restrictions. It is our opinion that the ICNIRP RL referred to workers motion in the fringe field are not indicative of the real occupational exposure. Gowland and Glover [35] stated that the ICNIRP guidelines are entirely based on their previous paper [24], which was the first in this research field. This was based on a very small study (twelve subjects). At the current state of knowledge, when the experience of vertigo is limited, only the static field limit would be accepted [35].

The weighted peak approach was also implemented for compliance assessment with the sensory and health stimulation limits. The results show the noncompliance with the EU Directive is confirmed by using the dB/dt computation.

The obtained results and the Glover’s considerations have to make us think that, because it is evident, the diffusion of ultra-high field MRI scanners involves, as a consequence, an increase of spatial and dB/dt time varying magnetic field when in motion. Consequently, the need to develop behavioral rules for workers and patients is highlighted such as in the case of ionizing radiation especially during surgery procedures. In fact, Glover [36] states that it is more difficult to prescribe regulatory limits for operators while the respect of behavioral rules may be more useful than a numerical action value. By insisting on these observations, it will be possible to gather a wide statistical pool of data to assist studies on health hazards. However, the need for defining methods for both risk and exposure evaluation remains an important research issue in which our methodology can be considered an exploratory study.

Particular attention must be paid to MRI manufacturer technicians, which are exposed to very high static magnetic fields and, during their work, to time varying magnetic fields.

In the meantime, we propose that the MRI workers should adopt specific behaviors especially when they move near the MRI gantry. In addition, the development of unfastened scanning beds equipped with a remote control panel could be a valid starting point. Actually, the MRI mobile bed would permit the healthcare staff to work far from the magnet gantry where the rate of change of the field does not cause vertigo and nausea.

## Figures and Tables

**Table 1:** Measurements conducted in different MRI sites using the TSMDs positioned on the MRI technicians’ Torso during routine patient positioning (Normal) simulating an emergency (Emergency).

	MRI Scanner	Duration (s)	$\left|\frac{dB}{dt}\right|$ Peak Value (mT/s) TSMD_1	$\left|\frac{dB}{dt}\right|$ Peak Value (mT/s) TSMD_2
Normal condition	0.35 T Siemens	112.0 ÷ 154.8	142 ± 8	152 ± 9
	1.5 T Philips	111.6 ÷ 300.0	510 ± 30	760 ± 50
	1.5 T Siemens	113.5 ÷ 159.4	470 ± 30	690 ± 40
	3.0 T Philips	110.6 ÷ 149.8	1900 ± 100	2650 ± 160
Emergency condition	0.35 T Siemens	10.4 ÷ 17.3	420 ± 20	470 ± 30
	1.5 T Philips	12.2 ÷ 19.4	2250 ± 120	2800 ± 170
	1.5 T Siemens	15.2 ÷ 25.4	1980 ± 110	2640 ± 160
	3.0 T Philips	18.6 ÷ 41.2	2830 ± 160	3540 ± 210

**Table 2:** Measurements conducted in different MRI sites using the TSMDs positioned on the MRI technicians’ Head during routine patient positioning (Normal) and when simulating an emergency (Emergency).

	MRI Scanner	Duration (s)	$\left|\frac{dB}{dt}\right|$ Peak Value (mT/s) TSMD_1	$\left|\frac{dB}{dt}\right|$ Peak Value (mT/s) TSMD_2
Normal condition	0.35 T Siemens	106.2 ÷ 152.0	196 ± 11	230 ± 14
	1.5 T Philips	108.8 ÷ 147.6	1930 ± 110	2640 ± 160
	1.5 T Siemens	118.2 ÷ 163.4	1727 ± 95	2640 ± 160
	3.0 T Philips	113.5 ÷ 140.1	2850 ± 160	3570 ± 210
Emergency condition	0.35 T Siemens	10.2 ÷ 17.4	470 ± 26	490 ± 30
	1.5 T Philips	12.5 ÷ 22.0	2530 ± 140	2910 ± 170
	1.5 T Siemens	13.6 ÷ 24.2	2780 ± 150	2920 ± 170
	3.0 T Philips	16.2 ÷ 28.2	3640 ± 200	5580 ± 330

**Table 3:** dB/dt peak value is extrapolated from the time derivative shown in Figure 3b.

MRI Scanner	Position	Measuring Conditions	Dosimeter	Duration (s)	$\left|\frac{dB}{dt}\right|$ Peak Value (mT/s)
3.0 T Philips	Torso	Normal	TSMD_1	2600	13900 ± 760

**Table 4:** dB/dt peak values and WP_L_ and WP_H_ indexes refer to routine procedures. Bold values indicate noncompliance with regulatory limits.

Dosimeter Position	MRI Scanner	Dosimeter	dB/dt Peak Value (mT/s)	WP_L_	WP_H_
Torso	0.35 T Siemens	TSMD_1	142 ± 8	0.72–0.89	0.12–0.23
		TSMD_2	152 ± 9	0.6–0.93	0.18–0.19
	1.5 T Philips	TSMD_1	510 ± 30	0.85–0.88	0.22–0.37
		TSMD_2	760 ± 30	0.75–0.95	0.12–0.26
	1.5 T Siemens	TSMD_1	470 ± 30	0.86–0.92	0.20–0.22
		TSMD_2	690 ± 40	0.89–0.92	0.14–0.23
	3.0 T Philips	TSMD_1	1900 ± 100	0.85–0.89	0.16–0.21
		TSMD_2	2650 ± 160	0.94–0.97	0.19–0.22
Head	0.35 T Siemens	TSMD_1	196 ± 11	0.68–0.87	0.07–0.11
		TSMD_2	230 ± 14	0.78–0.82	0.15–0.21
	1.5 T Philips	TSMD_1	1930 ± 110	0.91–0.94	0.38–0.41
		TSMD_2	2640 ± 160	0.92–**1.12**	0.32–0.55
	1.5 T Siemens	TSMD_1	1727 ± 95	0.86–0.91	0.28–0.36
		TSMD_2	2640 ± 160	0.92–**1.1**	0.32–0.54
	3.0 T Philips	TSMD_1	2850 ± 160	**1.15–1.3**	0.25–0.44
		TSMD_2	3670 ± 210	**1.07–1.42**	0.37–0.58

Note: Bold values indicate the non compliance with the regulatory limits.

**Table 5:** dB/dt peak values and WP_L_ and WP_H_ indexes refer to emergency simulations. Bold values indicate noncompliance with regulatory limits.

Dosimeter Position	MRI Scanner	Dosimeter	dB/dt Peak Value (mT/s)	WP_L_	WP_H_
Torso	0.35 T Siemens	TSMD_1	420 ± 20	0.21–0.32	0.09–0.12
		TSMD_2	470 ± 30	0.19–0.39	0.12–0.18
	1.5 T Philips	TSMD_1	2250 ± 120	0.75–0.88	0.06–0.14
		TSMD_2	2800 ± 170	0.92–0.94	0.29–0.46
	1.5 T Siemens	TSMD_1	1980 ± 110	0.85–0.88	0.26–0.52
		TSMD_2	2640 ± 160	0.90–0.93	0.27–0.50
	3.0 T Philips	TSMD_1	2830 ± 160	0.96–**1.1**	0.32–0.67
		TSMD_2	3540 ± 210	**2.24–4.1**	0.58–0.87
Head	0.35 T Siemens	TSMD_1	470 ± 26	0.27–0.38	0.12–0.16
		TSMD_2	490 ± 30	0.24–0.31	0.14–0.22
	1.5 T Philips	TSMD_1	2530 ± 140	0.91–0.95	0.35–0.64
		TSMD_2	2910 ± 170	**1.02–1.3**	0.42–0.77
	1.5 T Siemens	TSMD_1	2780 ± 150	0.99–**1.08**	0.37–0.75
		TSMD_2	2920 ± 170	**1.28–2.35**	0.35–0.81
	3.0 T Philips	TSMD_1	3640 ± 200	**2.6–4.2**	0.74–**1.21**
		TSMD_2	5580 ±330	**3.74–6.8**	**1.4–1.89**

Note: Bold values indicate the non compliance with the regulatory limits.

**Table 6:** dB/dt peak values and WP_L_ and WP_H_ indexes are reported. Bold values indicate noncompliance with regulatory limits.

Dosimeter Position	MRI Scanner	Dosimeter	dB/dt Peak Value (mT/s)	WP_L_	WP_H_
Torso	3.0 T Philips	TSMD_1	13900 ± 760	**8.9**	**3.2**

Note: Bold values indicate the non compliance with the regulatory limits.
